# The role of tissue elasticity in the differential diagnosis of benign and malignant breast lesions using shear wave elastography

**DOI:** 10.1186/s12885-020-07423-x

**Published:** 2020-09-29

**Authors:** Hui Yang, Yongyuan Xu, Yanan Zhao, Jing Yin, Zhiyi Chen, Pintong Huang

**Affiliations:** 1 Department of Ultrasound in Medicine, The Second Affiliated Hospital of Zhejiang University School of Medicine, Hangzhou, 310009 China; 2 Department of Ultrasound Medicine, Laboratory of Ultrasound Molecular Imaging, The Third Affiliated Hospital of Guangzhou Medical University, The Liwan Hospital of the Third Affiliated Hospital of Guangzhou Medical University, Guangzhou, 510000 Guangdong China

**Keywords:** Breast, Elastography, Shear wave elastography, Ultrasonography

## Abstract

**Background:**

Elastography is a promising way to evaluate tissue differences regarding stiffness, and the stiffness of the malignant breast lesions increased at the lesion margin. However, there is a lack of data on the value of the shear wave elastography (SWE) parameters of the surrounding tissue (shell) of different diameter on the diagnosis of benign and malignant breast lesions. Therefore, the purpose of our study was to evaluate the diagnostic performance of shell elasticity in the diagnosis of benign and malignant breast lesions using SWE.

**Methods:**

Between September 2016 and June 2017, women with breast lesions underwent both conventional ultrasound (US) and SWE. Elastic values of the lesions peripheral tissue were determined according to the shell size, which was automatically drawn along the edge of the lesion using the following software guidelines: (1): 1 mm; (2): 2 mm; and (3): 3 mm. Quantitative elastographic features of the inner lesions and shell, including the elasticity mean (E_mean_), elasticity maximum (E_max_), and elasticity minimum (E_min_), were calculated using an online-available software. The receiver operating characteristic curves (ROCs) of the elastographic features was analyzed to assess the diagnostic performance, and the area under curve (AUC) of each elastographic feature was obtained. Logistic regression analysis was used to predict significant factors of malignancy, permitting the design of predictive models.

**Results:**

This prospective study included 63 breast lesions of 63 women. Of the 63 lesions, 33 were malignant and 30 were benign. The diagnostic performance of E_max-3shell_ was the highest (AUC = 0.76) with a sensitivity of 60.6% and a specificity of 83.3%. According to stepwise logistic regression analysis, the E_max-3shell_ and the E_min-3shell_ were significant predictors of malignancy (*p* < 0.05). The AUC of the predictive equation was 0.86.

**Conclusions:**

SWE features, particularly the combination of E_max-3shell_ and E_min-3shell_ can improve the diagnosis of breast lesions.

## Background

Breast cancer is a global health burden and a leading cause of death in females worldwide [1]. Ultrasonography (US), as an adjunct technique for palpable or mammographically detected breast lesions, permits high sensitivity (typically≥90%) characterization of breast abnormalities [2, 3]. However, the US displays low specificity, thereby leading to unnecessary benign biopsies [4–6]. To improve the accuracy of the differential diagnosis of benign and malignant breast lesions, US elastography has been proposed as a non-invasive alternative. US elastography is an imaging technique that can be used to assess the stiffness or elasticity of breast masses, which is analogous to clinical palpation with US for a mass. The distinction between clinical palpation and elastography is that the former allows only a subjective judgment of the stiffness of a lesion, while elastography assesses tissue-specific differences in stiffness and/or elasticity, as lesions with an abnormal internal structure have altered elasticity [7–12] . For the assessment of breast lesions, two types of elastography are currently used, namely strain elastography (SE) and shear wave elastography (SWE). For SE, the major shortcomings are operator-dependency and a lack of quantitative information regarding the elasticity modulus. SWE provides quantitative values for the Young elastic modulus (in kilopascals) of tissues by imaging shear wave propagation, thus avoiding the shortcomings of SE [13, 14]. SWE has been shown to display high inter-and intra-observer reproducibility for both qualitative and quantitative parameters [15, 16]. In recent years, some studies had shown the stiffness of the tissue surrounding (shell) of the malignant breast lesions had been shown to be higher than that of benign breast lesions [17, 18]. To date, to our knowledge, the value of the SWE parameters of the different shell sizes on the diagnosis of benign and malignant breast lesions has not been assessed. In this prospective study, we hypothesized that these parameters might permit the differentiation between benign and malignant breast lesions. Therefore, the purpose of this study was to evaluate the diagnostic performance of shell elasticity in the diagnosis of benign and malignant breast lesions SWE.

## Methods

### Patients

This prospective study was approved by our institutional review board (IR001097). Written informed consent was obtained from all patients before examination.

**Fig. 1:**
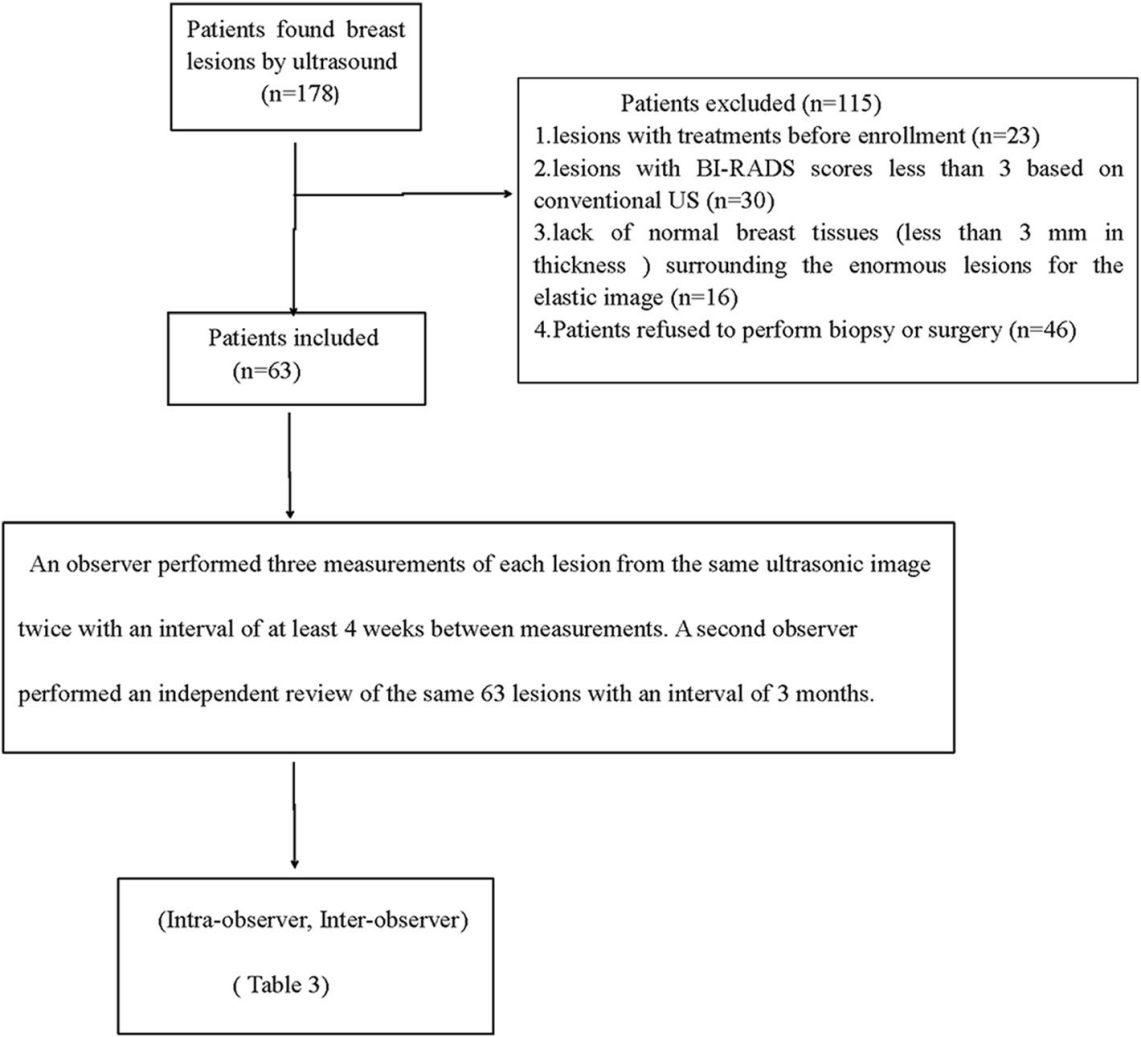
Flowchart for the selection of patients with breast nodules

From September 2016 to June 2017, a total of 178 consecutive patients with breast lesions who underwent the conventional US and SWE examination in our hospital, which were palpable by oncologists or visible on the conventional US, were enrolled in this study. The inclusion criteria were as follows: (1) breast lesions were palpable by an oncologist or were visible on the conventional US; (2) no treatment such as breast surgery, radiotherapy or chemotherapy was performed prior to enrollment. One hundred fifteen patients were excluded because of the following reasons: (1) lesions with treatments before enrollment; (2) lesions with BI-RADS scores less than 3 based on the conventional US; (3) lack of normal breast tissues (less than 3 mm in thickness) surrounding the enormous lesions for the elastic image and (4) no final histological results. A flowchart for the patients selection process was shown in Fig. 1. For evaluation, only 1 lesion with the highest BI-RADS category in each patient was selected. If multiple lesions were in the same BI-RADS category, the lesion with the largest diameter was selected.

### Ultrasound equipment

SWE and the conventional US were obtained using a Resona 7 diagnostic US system (Mindray Medical International, Shenzhen, China) equipped with an L14–5 linear transducer. The diagnostic system was equipped with a unique shell quantification toolbox, which was applied to measure the stiffness of the margin (0.5 ~ 9 mm) surrounding the lesion in 0.5 mm increments.

### Image evaluation

**Fig. 2:**
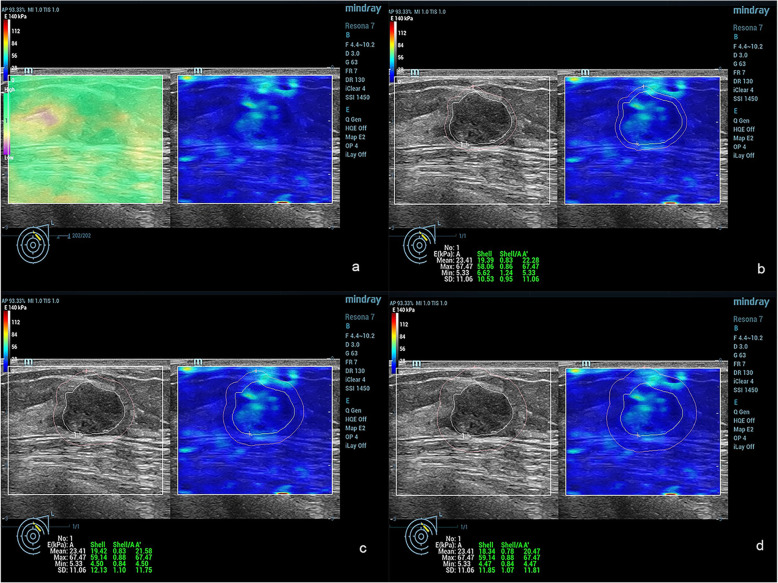
Fibroadenoma in a female patient. The E_max_ and E_min_ values of the breast lesion were 67.47 kPa and 5.33 kPa, respectively. **a**: SWE quality control with no obvious artifacts; **b**: The shell included 1 mm peripheral tissue around the breast lesion contour on the SWE image. The values of E_max-1shell_, E_mean-1shell_ and E_min-1shell_ were 58.06 kPa, 19.39 kPa and 6.62 kPa; **c**: The shell included 2 mm peripheral tissue around the breast lesion on the SWE image. The values of E_max-2shell_, E_mean-2shell_ and E_min-2shell_ were 59.14 kPa, 19.42 kPa, and 4.5 kPa; **c**: The shell included 3 mm peripheral tissue around the breast lesion on the SWE image. The values of E_max-3shell_, E_mean-3shell_, and E_min-3shell_ were 59.14 kPa, 18.34 kPa, and 4.47 kPa, respectively

**Fig. 3:**
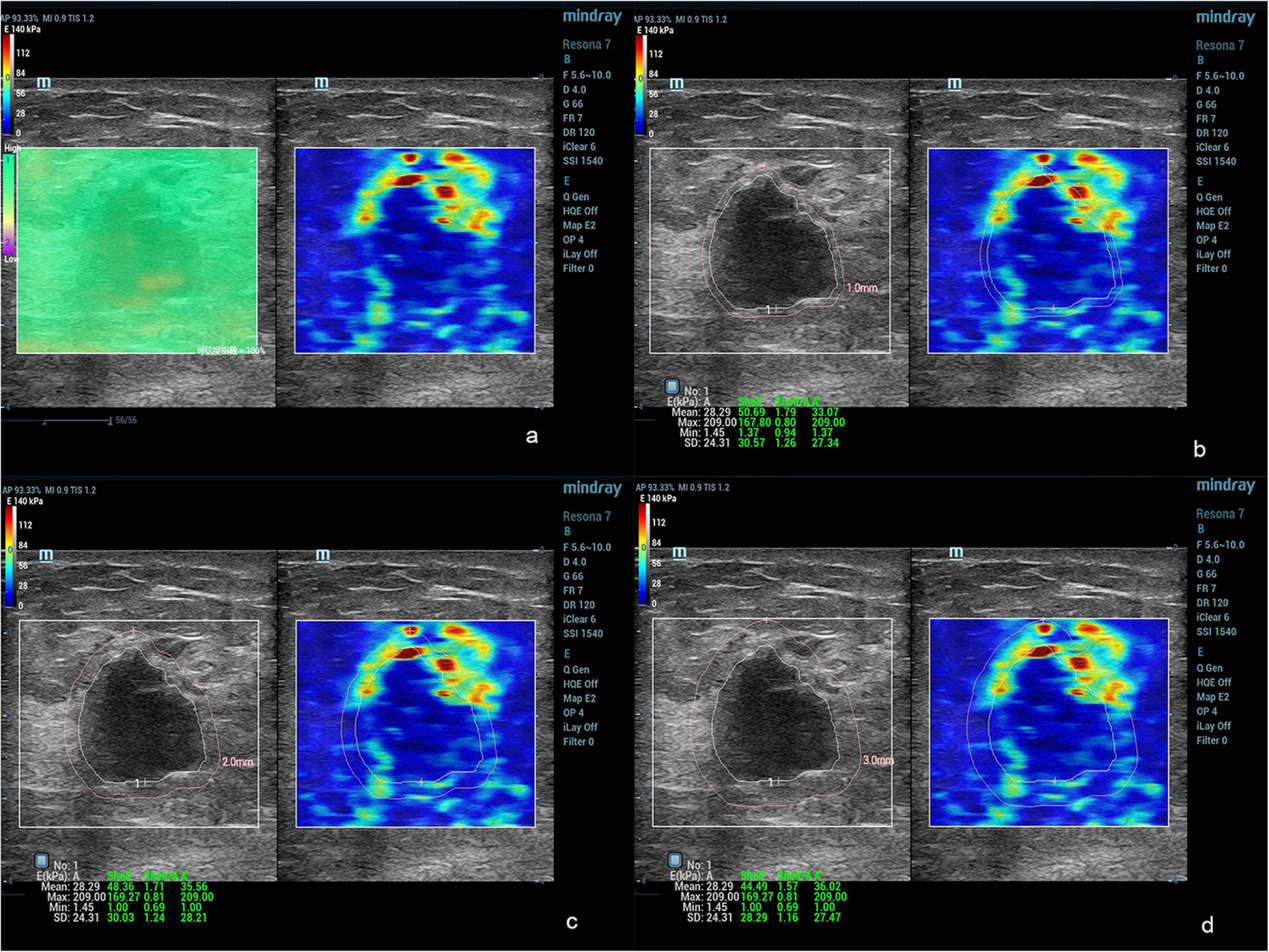
Infiltrating ductal carcinoma in a female patient. The E_max_ and E_min_ values of the breast lesion were 209.00 kPa and 1.45 kPa, respectively. **a**: SWE quality control with no obvious artifacts; **b**: The shell included 1 mm peripheral tissue around the breast lesion on the SWE image. The values of E_max-1shell_, E_mean-1shell_ and E_min-1shell_ were 167.8 kPa, 50.69 kPa and 1.37 kPa; **c**: The shell included 2 mm peripheral tissue around the breast lesion on the SWE image. The values of E_max-2shell_, E_mean-2shell_ and E_min-2shell_ were 169.27 kPa, 48.36 kPa, and 1.00 kPa; **c**: The shell included 3 mm peripheral tissue around the breast lesion on the SWE image. The values of E_max-3shell_, E_mean-3shell_, and E_min-3shell_ were 169.27 kPa, 44.49 kPa, and 1.00 kPa

Conventional US and SWE examinations were performed by a single radiologist (X.Y.Y.) with 20 years of experience in breast US. Quantitative SWE parameters were assessed by Y.H. (2 years of experience in breast US), and Z.Y.N. (3 years of experience in breast US) who were blinded to the BI-RADS score. Lesions for transverse and longitudinal US images were obtained in the supine position. Based on the gray-scale US image, all conventional US features of the lesions were assessed by using the terminology of the US BI-RADS lexicon. After a careful description of the lesions, a final BI-RADS assessment category was assigned. According to BI-RADS categories: BI-RADS 2 was benign; for BI-RADS 3, ultrasound of the breast revealed probable benign characteristics; BI-RADS 4a, 4b and 4c represented a low, moderate, and high suspicion of malignancy, respectively; BI-RADS 5 and BI-RADS 6 were highly suggestive of malignancy. According to the guidelines of the American Society of Radiology, a biopsy is recommended for breast lesions with BI-RADS 4a or higher. Follow-up is recommended for BI-RADS 3. The following steps were performed for correct elastic image acquisition: US examinations produced standard B-mode gray-scale images, and the lesions were placed in the center of the screen. During SWE measurements, the transducer was positioned perpendicular, and the pressure of the transducer was maintained to a minimum. Elastic images were obtained while patients held their breath. The reliability of the SWE images was assessed using a shear wave quality mode: the Quality Control Chart (QCC). When the color in the QCC was uniform, the SWE images were considered of high quality. When an imaging plane with the largest diameter of a breast lesion was located on conventional US images, a square region of interest (ROI) was set and adjusted to include the entire breast lesion and subcutaneous fat layer to the chest muscle layer for SWE acquisition. SWE images and B-mode conventional US were simultaneously displayed on a monitor. For SWE measurements, stiffness was quantified using the Young modulus (0–140 kPa). The dynamic model was selected, and quality control charts were simultaneously displayed to indicate good shear wave qualities and to ensure that no obvious artifacts were analyzed on the elastic modulus map. The ROI varied according to the size and shape of the breast lesion. Once the image stabilized, the ROI was drawn around the lesion. The ROI of the surrounding tissue was measured using the shell function according to shell size. A series of quantitative elastographic features of the inner lesion (E: E_mean_, E_max_, E_min_), the elastic mean of the shell size 1, 2, 3 mm (E_mean-shell_: E_mean-1shell_, E_mean-2shell_, E_mean-3shell_), the elastic maximum of the shell size 1, 2, 3 mm (E_max-shell_: E_max-1shell_, E_max-2shell_, E_max-3shell_), and the elastic minimum of the shell size (E_min-.shell_: E_min-.1shell,_ E_min-.2shell_, E_min-.3shell_) were calculated (Figs. 2 and 3).

### Observer variability evaluation

Intra-observer agreement was assessed by a radiologist (Y.H) who performed three measurements of each lesion from the same ultrasonic image twice with an interval of at least 4 weeks between measurements. To assess inter-observer variability, a second observer (Z.Y.N), who was blinded to the previous US and histopathological results, performed an independent review of the same 63 lesions with an interval of 3 months. Agreements between the two measurements by the different observers were evaluated.

### Histopathological examination

Histopathological examination was used as the reference standard for all patients. Histopathological diagnosis was performed by an experienced pathologist (≥ 15 years’ experience) who was blinded to the ultrasound results.

### Statistical analysis

Statistical analyses were performed using SPSS, version 17.0 (SPSS, Chicago, IL, USA). ROC analysis was performed by using MedCalc for Windows, version 13.1.2.0 (MedCalc Software, Mariakerke, Belgium). Optimal cutoff values were determined through the Youden index (maximum of sensitivity + specificity - 1). The independent samples t-test was used to compare the quantitative SWE values. The McNemar test was employed for the paired comparison of proportions (sensitivity, specificity, positive prediction, and negative prediction values). A step-wise multivariate logistic regression analysis was used to identify risk factors and risk models for malignancy. Intraclass correlation coefficients (ICCs) were used to assess intra-and inter-observers. A *p* value less than 0.05 was considered statistically significant differences.

## Results

### Study population

A total of 63 patients with breast lesions were enrolled in this study. Among them, 33 lesions were malignant and 30 were benign. The age of the included patients ranged from 19 to 86 years, with an average age of 46.8 years. The mean age of the benign and malignant patients included in our study was 38.5 ± 14.7 years (range, 19–86 years) and 54.4 ± 12.5 years (range, 30–80 years), respectively. The maximal diameter of the lesions from the conventional US was 20.0 ± 8.6 mm (range: 5.1–51.3 mm). The mean diameter ± SD of malignant and benign nodules were 20.3 ± 7.5 mm and 19.6 ± 9.7 mm, respectively. No significant differences were observed in the size of the benign and malignant breast lesions (*p* > 0.05). Ultrasound-guided core needle biopsies were performed in all lesions, and 59 lesions underwent surgery. From pathological assessments, the malignant lesions included mucinous carcinoma (*n* = 1), infiltrating ductal carcinoma (*n* = 25), invasive lobular carcinoma (*n* = 1), papillary carcinoma (*n* = 1), and ductal carcinoma in situ (*n* = 5). Benign diagnoses were as follows: fibroadenoma (*n* = 18), fibroadenomatous hyperplasia (*n* = 3), papilloma (*n* = 3), inflammation (*n* = 2), and adenosis (*n* = 4). Histopathological results of the benign and malignant tumors are summarized in Table 1. For the conventional ultrasound BI-RADS category, the numbers of category 3, 4a, 4b, 4c, 5, and 6 cases were 10/63 (15.9%), 11/63 (17.5%), 11/63 (17.5%), 12/63 (19.0%), 13/63 (20.6%), and 6/63 (9.5%), respectively. The malignancy rates were 10% (1/10) for category 3, 0.0% (0/11) for category 4a, 36.4% (4/11) for category 4b, 75.0% (9/12) for category 4c, 100.0% (13/13) for category 5, and 100.0% (6/6) for category 6. Category 4a had the lowest likelihood of malignancy, while categories 5 and 6 had the highest likelihood. The optimal cutoff was between category 4a and category 4b.

**Table 1:** Summary of pathologic findings and performance of conventional ultrasound

Histopathological results	Conventional US BI-RADS category						
	No of lesions (%)	3	4A	4B	4C	5	6
Benign	30 (47.6)						
Fibroadenoma	18 (60.0)	7	9	1	1	0	0
Fibroadenomatous Hyperplasia	3 (10.0)	1	0	1	1	0	0
Papilloma	3 (10.0)	1	0	2	0	0	0
Inflammation	2 (6.7)	0	1	1	0	0	0
Adenosis	4 (13.3)	0	1	2	1	0	0
Malignant	33 (52.4)						
Mucinous Carcinoma	1 (3.0)	0	0	1	0	0	0
Infiltrating ductal carcinoma	25 (75.8)	1		3	5	11	5
Invasive lobular carcinoma	1 (3.0)	0	0	0	0	1	0
Papillary carcinoma	1 (3.0)	0	0	0		1	0
Ductal carcinoma in situ	5 (15.2)	0	0	0	4		1

#### Diagnostic performance of the quantitative SWE features

##### Diagnostic performance of SWE parameters of the shell (E_shell_)

**Fig. 4:**
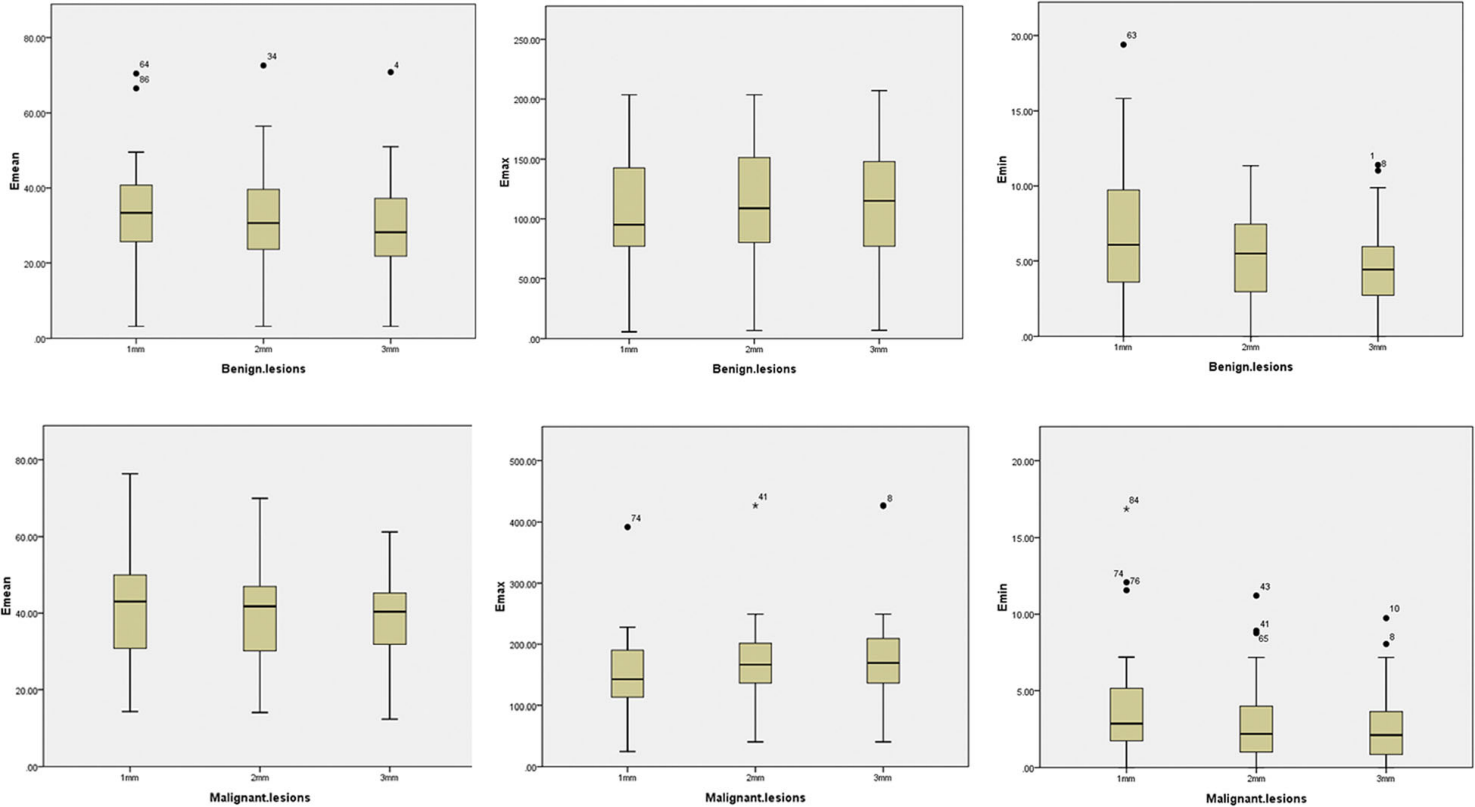
Box and whisker plots of the mean elasticity, maximum elasticity, and minimum elasticity values at 1, 2, and 3 mm of the shell in both malignant (**a**) and benign (**b**) lesions

The elastographic values of the shell (E_mean-shell_, E_max-shell_ and E_min-shell_) significantly differed between benign and malignant breast lesions. The E_min-shell_ values were significantly lower in malignant lesions compared to benign lesions (*p* < 0.05). The values of E_max-3shell_ and E_max-2shell_ for invasive breast carcinomas were significantly higher than those of non-invasive carcinomas (*p* < 0.05). The elastographic values of the shell were shown in Table 2, and the results are depicted by box plots (Fig. 4) for malignant and begin lesions. Amongst the E_shell_ parameters for the lesions with BI-RADS scores of 3 or greater, E_max-3shell_ had the highest AUC: 0.76 (95% CI 0.63, 0.86) with a sensitivity of 60.6%, a specificity of 83.3%, positive predictive values of 80.0%, and negative predictive values of 65.8%. No significant differences were observed in the AUCs amongst the elastic parameters. The specificity and positive predictive values of the E_max-3shell_ were higher compared to that of other elastic parameters (*p* < 0.05).

**Table 2:** Quantitative elastic features of the inner and peripheral tissue of the lesions

	Benign	Malignant						
	[Mean] (kPa)	[Mean] (kPa)	Sensitivity (%)	Specificity (%)	PPV (%)	NPV (%)	AUC (95% CI)	*p* Value
E_max_	115.81	154.72	66.7	70	71.0	65.6	0.68 (0.56, 0.80)	0.037
E_min_	6.06	3.05	87.9	53.3	67.4	80.0	0.71 (0.58, 0.82)	0.004
E_max-3shell_	112.45	169.74	60.6	83.3	80.0	65.8	0.76 (0.63, 0.86)	0.000
E_mean-3shell_	29.77	38.84	84.8	60.0	70.0	78.3	0.73 (0.61, 0.84)	0.006
E_min-3shell_	4.89	2.68	78.8	66.7	72.0	74.0	0.73 (0.61, 0.84)	0.002
E_max-2shell_	112.51	167.07	78.8	66.7	72.2	74.1	0.75 (0.62, 0.85)	0.001
E_mean-2shell_	32.28	37.15	60.6	83.3	80.0	65.8	0.70 (0.58, 0.81)	0.014
E_min-2shell_	5.41	3.08	72.7	73.3	75.8	73.3	0.73 (0.60, 0.83)	0.002
E_max-1shell_	107.27	151.15	66.7	70.0	71.0	65.6	0.70 (0.57, 0.81)	0.004
E_mean-1shell_	33.67	41.92	57.6	80.0	76.0	63.2	0.70 (0.55, 0.79)	0.017
E_min-1shell_	6.51	3.99	63.6	76.7	75.0	65.7	0.70 (0.57, 0.80)	0.026

*Abbreviations*: *PPV* positive predictive value, *NPV* negative predictive value, *AUC* the area under the receiver operating characteristic curve

*p*-Value indicates that there is significantly different between those values of overall benign and malignant breast lesions

##### Diagnostic performance of the SWE parameters of the inner lesions

The E_max_ and E_min_ values significantly differed between benign and malignant breast lesions. The E_min_ values were significantly lower in malignant lesions compared to benign lesions (*p* < 0.05). The AUC of the E_max_ and E_min_ were 0.68 (95% CI 0.56, 0.80) and 0.71 (95% CI 0.58, 0.82) for the lesions with BI-RADS scores of 3 or greater. No significant differences were observed between the AUCs of the E_max_ and E_min_. The sensitivity, specificity, positive prediction values, and negative prediction values of E_max_ and E_min_ were 66.7, 70, 71.0, 65.6, and 87.9%, 53.3, 67.4, 80%, respectively. The AUC, sensitivity, specificity, positive prediction value (PPV), negative prediction value (NPV) of the E, and E_shell_ were summarized in Table 2.

### Multivariate logistic regression analysis

**Fig. 5:**
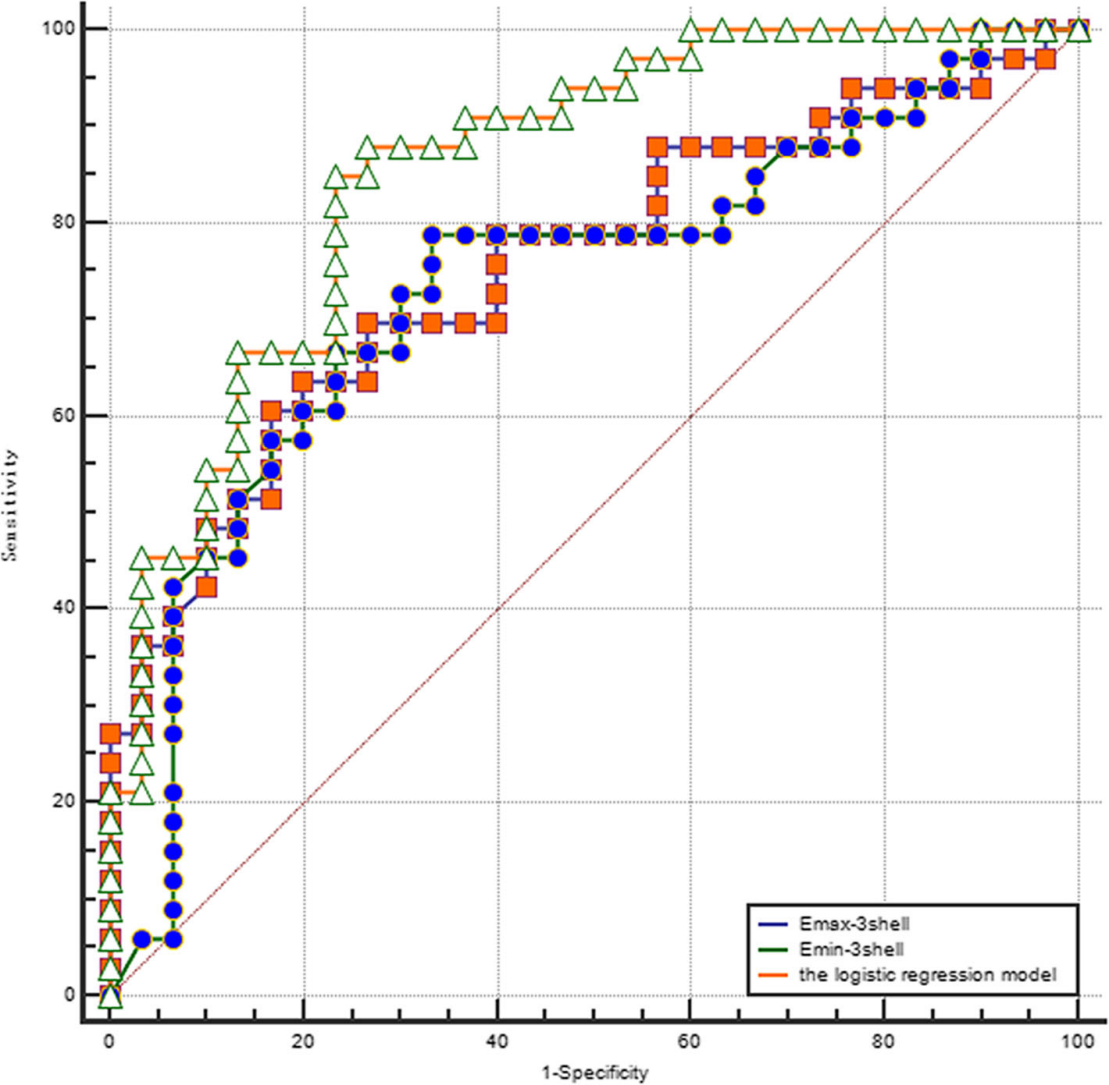
Receiver operating characteristic curves of the E_max-3shell_ and E_min-3shell_, and logistic regression model values for analyzing the diagnostic performance (AUC of the E_max-3shell_, 0.76; AUC of the E_min-3shell,_ 0.73; AUC of the logistic regression model values, 0.86)

Univariate analysis showed that the E_shell_, E_max_ and E_min_ values significantly differed for the prediction of benign and malignant breast lesions. The elastic parameters were further analyzed using step-wise multivariate logistical regression, and upon logistical regression analysis, the E_max-3shell_ and E_min-3shell_ were significant independent predictors of malignancy with Odds Ratios (OR) of 1.02 (95% CI 1.009–1.037; *p* < 0.05) and 0.65 (95% CI 0.494–0.853; *p* < 0.05), respectively. The stability of multivariate logistic regression models was tested by Cross-Validation in Python, the training/testing split is 80%/ 20%, we assigned 80% of patients as the training set, and the remaining 20% used the test set, this procedure was repeated for twice, the recall (recall = TP/TP + FN) were 0.83 and 0.88 respectively, the AUC were 0.85 and 0.84 respectively, the result indicated that the predictive model is reliable. The AUC of the predictive model was significantly higher compared to that of the E_max-3shell_ and E_min-3shell_ (both *p* < 0.05). Upon comparison of the AUC of E_max-3shell,_ E_min-3shell_ and the predictive model, significant differences were observed in the AUC (Fig. 5). The logistic regression model significantly improved the diagnostic performance compared to the E_max-3shell_ and E_min-3shell_ alone, with a sensitivity and specificity of 84.9 and 76.7%, respectively.

### Observer agreements of SWE features

The ICC was measured on a scale of 0 to 1. The observer agreement was divided into three grades: slight agreement (0.01 < ICC < 0.40), moderate agreement (0.40 < ICC < 0.75), and almost perfect agreement (0.75 < ICC < 1). In our study, the intra-observer agreement and inter-observer agreements were almost perfect. The result were shown in Table 3.

**Table 3:** Interobserver and Intraobserver variability of SWE Measurements in Breast Lesions

	Interobserver Variability	Intraobserver Variability
	ICC	ICC
E_mean-3shell_	0.83	0.90
E_max-3shell_	0.88	0.99
E_min-3shell_	0.89	0.92
E_mean-2shell_	0.83	0.90
E_max-2shell_	0.88	0.98
E_min-2shell_	0.88	0.95
E_mean-1shell_	0.82	0.90
E_max-1shell_	0.87	0.98
E_min-1shell_	0.81	0.97
E_max_	0.88	0.98
E_min_	0.81	0.98

## Discussion

In previous studies, it has been shown that qualitative and quantitative SWE parameters can improve the differentiation of benign and malignant breast lesions when employed as an additional sonographic technique [19, 20]. Some studies had also reported that the peripheral tissue of malignant breast tumors is typically stiffer than inner lesions due to the presence of abnormal stiff collagen associated with cancer fibroblasts, and the infiltration of cancer cells into peri-lesions of the tissue [21–23]. Zhou et al. [24] evaluated the presence of the stiff rim sign at 180 kPa, and at less than 180 kPa, the result showed that for display settings ≤180 kPa, the stiff rim sign had a higher potential to differentiate between breast lesions. Tozaki and Fukuma [25] had proved Color patterns of 3-dimensional (3D) SWE were useful in the differential diagnosis of breast lesions. Moreover, Chen et al. [26] evaluated 3 views reconstructed by 3D SWE with emphasis on that of transverse, sagittal, and coronal planes. The result revealed that 3D SWE color patterns significantly increased diagnostic accuracy, with the coronal plane of the highest value. However, these studies focused on the stiff rim sign of SWE, without emphasis on the diagnostic performance of different sizes of surrounding tissue (shell) elasticity in the diagnosis of benign and malignant breast lesions. In this study, we applied a shell quantification toolbox feature and proposed quantitative measurements according to the diameter of the shell (1, 2 & 3 mm). The color range was displayed at 0–140 kPa. The results showed that the elastographic values of the shell (E_shell_) significantly differed between benign and malignant breast lesions. Among the elastic parameters, E_max-3shell_ had a higher AUC (0.76), while no significant differences were observed in the AUCs among the elastic parameters (*p* > 0.05). Park et al. [27] compared the peritumoral stroma (PS) tissue stiffness of benign and malignant breast lesions by setting multiple rounds 2 mm ROIs in a linear arrangement onto the inner tumor, tumor-stroma border, and PS. The results indicated that malignant tumors showed a “rapid increase–decrease” pattern and that the maximum elasticity values were observed within proximal PS, which was about 2 ~ 4 mm from the edge of the tumor. The result was similar to our findings. For this phenomenon, one explanation would be that the peritumoral stiffness was increased because of a desmoplastic reaction or infiltration of cancer cells into the stroma. Another explanation would be that attenuation of the energy of the shear wave in the peritumoral region of the lesion might cause a low shear wave amplitude within the malignant lesion [22, 28]. In previous studies, the E_max_ and E_mean_ were the best-performing SWE parameters for differentiating malignant and benign breast lesions [29–31]. In this study, the E_mean_ did not significantly differentiate malignant and benign lesions. The E_mean_ is equal to the sum of all elasticity values of each pixel divided by the number of pixels within the ROI. The elasticity value is influenced by the size of the ROI [32], which was created manually according to the lesion size using the Mindray ultrasound system. The relative differences in ROI may account for the discrepancies between the studies. Xiao et al. [33] showed that for the logistic regression models, combining the SE features significantly improved diagnostic performance compared to B-mode US. In this study, we proposed a more comprehensive approach, including the analysis of lesion stiffness and surrounding tissue stiffness incorporated into the logistic regression model to discriminate between benign and malignant breast lesions. Univariate analysis showed that the E_max-3shell_ and E_min-3shell_ could significantly predict malignant breast lesions. The reliability of the logistic regression model that combined E_max-3shell_ and E_min-3shell_ was confirmed by the AUC of 0.86, which was higher than the individual AUC of the E_max-3shell_ and E_min-3shell_. Compared to the AUC of the E_max-3shell_, E_min-3shell_ and the predictive model, significant differences were observed. The logistic regression model had a higher diagnostic performance for benign and malignant breast lesions. Using the cut-off value of E_max-3shell_ (156.96 kPa) and E_min-3shell_ (3.99 kPa) as discriminative parameters, the negative predictive values for malignancy were only 65.79 and 66.67%, respectively. The logistic regression analysis showed that the negative predictive value was 71.9%, which was improved. Vinnicombe et al. [34] demonstrated that in situ ductal carcinomas (DCIS) were likely to display benign shear wave features. However, in our study, only a single (20%; 1/5) DCIS showed false-negative findings by using the logistic regression model. This phenomenon showed that the logistic regression model might contribute to an improvement in diagnostic accuracy for DCIS. However, since the number of cases included in this study is small, more cases will be needed for verification in the future. While in this study, 8 malignant lesions were still false-negatives (24.2%; 8/33), in 8 of the false-negative cases, 4 had a lesion size ≤15 mm and 1 had a lesion size ≤10 mm. Previous studies had shown that malignancies ≤15 mm and/or ≤ 10 mm tend to show benign features leading to false results [22].

There were some limitations to this study. Firstly, a small sample size is a limitation of the present study. Breast nodules are common disease in clinical, a total of 178 consecutive patients with breast lesions who underwent the conventional US and SWE examination were selected in this study. However, for the exclusive reasons, only 63 patients were finally enrolled in this study. Secondly, we did not assess the diagnostic performance of ultrasound features combined with BI-RADS, meanwhile, lesions with BI-RADS scores less than 3 based on the conventional US were excluded in this study, which may result in selection bias. Finally, factors influencing the elastic characteristics of the surrounding tissues, including lesion depth, breast density and pre-compression, were not evaluated.

## Conclusion

E_shell_ values are highly correlative to malignant breast lesions. SWE features, particularly the combination of E_max-3shell_ and E_min-3shell_ can improve the differentiation of breast lesions. The logistic regression model enabled the correct differentiation of benign and malignant breast lesions with a sensitivity of 84.9% and a specificity of 76.7%. The diagnostic performance of this model exceeded that of the elastographic parameters of E_shell_ and E alone when evaluating benign and malignant breast lesions.

## Data Availability

The datasets used and/or analyzed in the current study are available from the corresponding author upon request.

## Abbreviations

SWE: Shear wave elastography
AUC: The area under the receiver operating characteristic curve
US: Ultrasonography
ACR: The American College of Radiology
SE: Strain elastography
ROI: Region of interest
ICCs: Intraclass correlation coefficients
OR: Odds ratio
DCIS: Ductal carcinoma in situ
QCC: Quality control chart
SD: Standard deviation
TP: True positive
FP: False positive
TN: True negative
FN: False negative
